# RNAi of *vATPasea* Affects Survival and Larval-Pupal Development in *Plutella xylostella*

**DOI:** 10.3390/insects16101054

**Published:** 2025-10-16

**Authors:** Xuetao Yu, Jinhua Luo, Lin Lu, Li Zhu, Siyuan Wang, Kang Yang, Xia Wan, Yuhua Wu, Boboev Akmal, Gang Wu, Xiaohong Yan, Chenhui Shen

**Affiliations:** 1 Key Laboratory of Agricultural Genetically Modified Organisms Traceability, Oil Crops Research Institute of Chinese Academy of Agricultural Sciences, Ministry of Agriculture and Rural Affairs, Wuhan 430062, China; xuetao.yu@outlook.com (X.Y.); ll15966192116@163.com (L.L.); zhuli49751@163.com (L.Z.); a148151153158@163.com (S.W.); yawa521@gmail.com (K.Y.); wanxia@oilcrops.cn (X.W.); wuyuhua@oilcrops.cn (Y.W.); wugang@caas.cn (G.W.); 2 Enshi Tujia and Miao Autonomous Prefecture Academy of Agricultural Sciences, Enshi 445000, China; chw63@126.com; 3 Department of Chemical Technology of Silicate Materials and Rare, Tashkent Institute of Chemical Texnology, Tashkent 100011, Uzbekistan; akbob16@gmail.com

**Keywords:** *Plutella xylostella*, RNA interference, introduction, *PxvATPasea*

## Abstract

**Simple Summary:**

Relatively little attention has been given to genes encoding V_0_ subcomplex subunits. In the study, we identified *PxvATPasea* specifically and evaluated expression profiles of *PxvATPasea* across developmental stages and among tissues. By injecting two doses of ds*PxvATPasea* (800 ng or 1200 ng) in *Plutella xylostella*, we showed that *PxvATPasea* is a potential molecular target gene and RNAi efficiencies worked in a dose-dependent way.

**Abstract:**

The diamondback moth, *Plutella xylostella*, is a major pest of brassica vegetables and oilseed crops, posing a serious threat to China’s grain and oil production. RNA interference (RNAi) has been developed as an efficient strategy to control pests. In this study, the effects of RNAi on *P. xylostella* were evaluated by injecting two doses of synthesized ds*PxvATPasea*. The transcripts of *PxvATPasea* were widely transcribed during different developmental stages from egg to adult. They were abundantly expressed in the hindgut and Malpighian tubules, compared with other tissue types. Introduction of 800 ng ds*PxvATPasea* in the fourth-instar larvae greatly reduced corresponding mRNA levels by 3.1 and 1.4 times on day 2 and 3, respectively, causing 66.6% mortality and 33.4% treated larvae pupated. Silencing *PxvATPasea* by injecting 1200 ng dsRNA significantly decreased the expression level by 5.0 and 2.0 times on the second and third day, leading to 79.2% larval lethality and 20.8% depleted larvae pupated. Moreover, introducing 800 ng or 1200 ng ds*PxvATPasea* finally reduced larval fresh weight by 22.1% and 28.8%, respectively. The results indicated that the silencing efficiency of *PxvATPasea* worked in a dose-dependent way. Consequently, *PxvATPasea* is a potential molecular target gene. Our findings will facilitate the application of RNAi technology to manage *P. xylostella.*

## 1. Introduction

The diamondback moth, *Plutella xylostella*, is one of the primary lepidopteran pests attacking brassica vegetables and oilseed crops, causing monetary losses of around US $4–5 billion worldwide [1,2]. At present, chemical pesticides are applied mainly to manage this pest, such as chlorantraniliprole, chlorpyrifos, and indoxacarb [3]. However, owing to the rapid development of resistance, it is more and more difficult to control its field populations based on insecticides [2]. Thus, it seems much more urgent and necessary to develop an efficient strategy to manage the population of *P. xylostella*.

RNA interference (RNAi) is an efficient molecular mechanism for suppressing the expression of post-transcriptional target genes triggered by double-stranded RNA (dsRNA), which can be used to develop an environmentally friendly and reduced-risk insect pest management approach [4,5,6,7]. In recent years, numerous research have demonstrated that RNAi-based integrated pest management works well in various insects [7,8,9,10,11,12,13]. For example, use plant-mediated RNA interference strategy to control *Frankliniella occidentalis* [9] and *Apolygus lucorum* [10]; RNAi silencing protease genes can affect mortality in *Helicoverpa armigera* [12]; continuous ingestion of ds*LdRan* can kill the larvae and adults of *Leptinotarsa decemlineata* [7]; knockdown of *HvUAP* impairs larval growth in *Henosepilachna vigintioctopunctata* [13]. Currently, silencing target genes *PxPiwi* [14] and *PxTH* [15] in *P. xylostella* affected larval development. Thus, it seems practicable to apply RNAi-based methods to manage population of *P. xylostella*.

Vacuolar-type H^+^-ATPase (vATPase) is a proton translocating pump localized in the internal membranes of eukaryotes, functioning by hydrolyzing ATP to ADP and phosphate to pump protons across membranes [16,17,18,19]. The structures of vATPases are evolutionarily conserved in eukaryotes, consisting of two subcomplexes, V_1_ and V_0_ [20,21]. The V_1_ subcomplex contains eight different subunits (A through H), with stoichiometry of A_3_B_3_CDE_3_FG_3_H in *Manduca sexta* and *Saccharomyces cerevisiae*, which is related to ATP hydrolysis. The V_0_ subcomplex consists of five different subunits (a_1_d_1_c_4–5_c′_1_c″_1_) and accessory proteins, which can drive transmembrane proton movement [21,22,23,24,25,26].

Currently, relatively little attention has been given to genes encoding V_0_ subcomplex subunits, and only two research have verified that depletion of *vATPasea* was lethal [27,28]. In *Drosophila melanogaster*, P-element insertions in *vha100-2* are lethal [27]. In *H. vigintioctopunctata*, knockdown of *HvvATPasea* in the third-instar larvae caused 100% larval lethality [28]. It is vital to determine whether silencing genes encoding V_0_ subcomplex subunits results in severe defective phenotypes in other insects. In addition, compared to Bt-transgenic *Brassica napus*, expression of *CHS1 hp*RNA in transgenic *B. napus* shortened the mortality time in *P. xylostella* [1]. Therefore, RNAi-based control of *Plutella xylostella* populations by feeding them dsRNA targeting *PxvATPasea* holds promise for future development.

In the present study, *PxvATPasea* was chosen as the target gene to assess the possibility of management methods for *P. xylostella*. Specifically, we (i) cloned, aligned, and phylogenetically analyzed the *vATPasea* gene in *P. xylostella*; (ii) evaluated expression profiles of *PxvATPasea* across developmental stages and among tissues; (iii) assessed the RNAi efficiency by measuring the suppression of *PxvATPasea* expression after treatment with synthesized ds*PxvATPasea* in vitro; and (iv) examined the effects on larval weight, survival rate, pupation rate, and emergence rate following injection of ds*PxvATPasea*.

## 2. Materials and Methods

### 2.1. Insect Rearing

*P. xylostella* was collected from *B. napus* in Haidong City, Qinghai Province, China. The larvae of *P. xylostella* were fed on fresh brassica leaves at 26 ± 1 °C, 16 h:8 h photoperiod, and 60% ± 5% relative humidity. The adults were reared using 10% honey solution as food.

### 2.2. Molecular Cloning

The putative *PxvATPasea* was obtained from the genome and transcriptome data of *P. xylostella* [29,30]. The correctness of the sequence was proven by polymerase chain reaction (PCR) using primers in Table S1 and Figure S1. The accession number of sequenced cDNA was XP_048479200.1.

Phylogenetic analysis of *PxvATPasea* and other *vATPasea* sequences downloaded from NCBI (https://www.ncbi.nlm.nih.gov/, accessed on 18 April 2024) was conducted using MEGA 5.0 software (https://sourceforge.net/projects/mega5/, accessed on 18 April 2024) and the neighbor-joining method, with 1000 bootstrap replications.

### 2.3. Synthesis of dsRNA Molecules

The two cDNA fragments targeting PxvATPasea and enhanced green fluorescent protein were amplified by PCR using special primers containing the T7 promoter sequence (Table S1, Figure S1). To ensure the specificity and efficacy of the dsRNA, the target sequences by BioEdit 7.0 software were subjected to BLASTN analysis against the *P. xylostella* transcriptome and evaluated using a web-based prediction tool (https://www.dsrna-engineer.cn, accessed on 18 April 2024) for on-target and off-target sites. Regions with a high density of on-target sites were selected while avoiding potential off-target sequences, thereby excluding any segments with ≥20 bp identical matches that could lead to off-target effects (Figure S2). dsRNA was then synthesized using the MEGAscript T7 High Yield Transcription Kit (Ambion, Austin, TX, USA), according to the manufacturer’s instructions. The reaction mixture contained 2 μL of T7 Express Enzyme Mix (Promega, Beijing, China), 10 μL of RiboMAX™ Express T7 2× Buffer (Promega, Beijing, China), and 1 μg of DNA template and was incubated at 37 °C for 2–6 h, followed by 70 °C for 10 min, and slowly cooled to room temperature (approximately 20 min) for dsRNA annealing. The DNA template and single-stranded RNA were removed by treatment with DNase and RNase A, respectively, and the dsRNA product was purified using a gel extraction kit (Omega Bio-tek, Norcross, GA, USA). The yield of dsRNA was determined by measuring absorbance at 260 nm with a Nanodrop 1000 spectrophotometer (Thermo Fisher Scientific, Waltham, MA, USA), and its integrity was verified by agarose gel electrophoresis. The purified dsRNA was aliquoted and stored at −80 °C until use.

### 2.4. Introduction of dsRNA

The same method as previously described was used to inject dsRNA [31,32,33]. Briefly, newly molted fourth-instar larvae were selected for microinjection. Borosilicate microcapillaries were pulled using a PC-10 puller (Narishige, Tokyo, Japan). A volume of 200 nL containing one of two dsRNA doses (800 ng or 1200 ng in total, based on references [34]) was delivered into the larval hemolymph using a microinjection system from World Precision Instruments (Sarasota, FL, USA). Larvae injected with ds*GFP* served as the negative control. A group of eight injected larvae constituted one replicate, and each dsRNA treatment was repeated six times. After injection, larvae were transferred to individual plastic rearing boxes and fed with fresh rape leaves. For sampling, three replicates were collected 2 and 3 days post-injection for qRT-PCR analysis to assess RNAi efficiency. Another three replicates were maintained for a 3-week observation period to monitor defective phenotypes, changes in larval weight, survival rate, pupation rate, and emergence rate.

### 2.5. Real-Time Quantitative PCR (qRT-PCR)

RNA templates of samples were collected to analyze the temporal expression profiles and tissue transcription patterns. Moreover, for testing the effects of the treatments, total RNA was extracted from treated larvae by the TRIzol reagent (YiFeiXue Tech, Nanjing, China). Each sample contained eight individuals and was repeated three times. Quantitative real-time PCRs (qRT-PCR) were conducted to assess the transcript expression levels according to the previously described method [35]. The qRT-PCR was performed with three biological and technical replicates. The relative mRNA expression levels were analyzed using 2^−ΔΔCt^ method.

### 2.6. Data Analysis

We used The SPSS Statistics 27 software for Windows (Chicago, IL, USA) for the statistical analyses. The averages (±SE) were used to analyze variance with the Tukey–Kramer test. Survival curves were analyzed using a log-rank test (Mantel–Cox, 95% CI) in GraphPad Prism software version 8.0.

## 3. Results

### 3.1. Identification of PxvATPasea

A putative full-length cDNA encoding *PxvATPasea* was obtained in *P. xylostella* by mining transcriptome data. The cDNA was comprised of a 2520 bp complete open reading frame encoding 839 amino acid residues (Figure S1).

**Figure 1:**
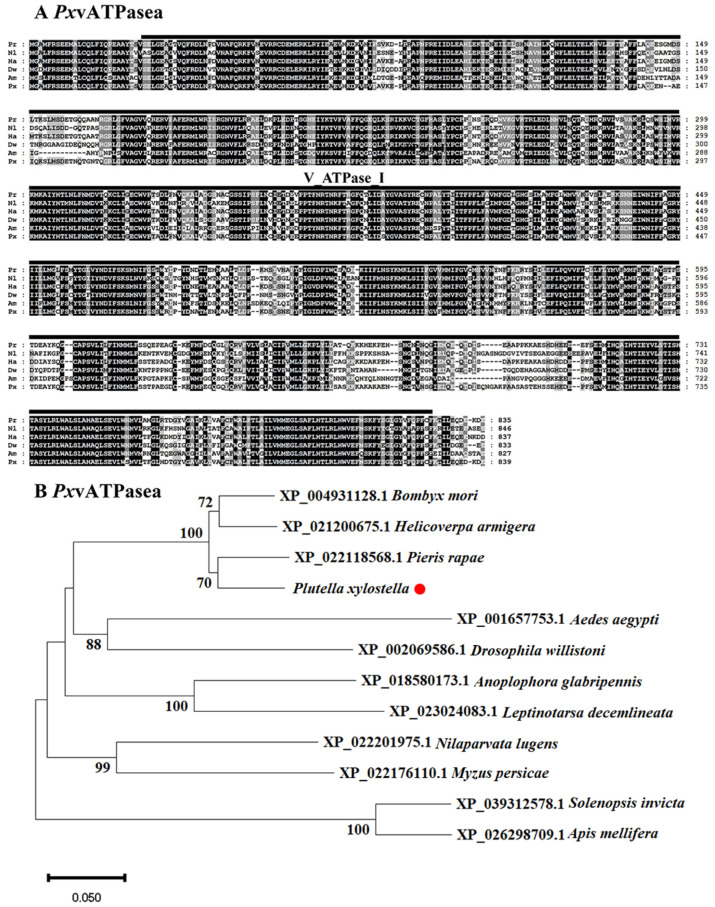
Alignment (**A**) and phylogenetic analysis (**B**) of vATPase subunits a (*PxvATPasea*) derived from *Plutella xylostella*. (**A**) The proteins originate from *Pieris rapae* (*Pr*), *Nilaparvata lugens* (*Nl*), *Helicoverpa armigera* (*Ha*), *Drosophila willistoni* (*Dw*), *Apis mellifera* (*Am*), and *Plutella xylostella* (*Px*). Increasing background intensity (from light to dark) indicates an increase in sequence similarity. Gaps are introduced to permit alignment. (**B**) vATPasea proteins are from four Lepidoptera, *Bombyx mori*, *Pieris rapae*, *Plutella xylostella*, and *Helicoverpa armigera*; two Hymenoptera, *Apis mellifera* and *Solenopsis Invicta*; two Diptera, *Aedes aegypti* and *Drosophila willistoni*; two Hemiptera, *Nilaparvata lugens* and *Myzus persicae*; and two Coleoptera, *Leptinotarsa decemlineata* and *Anoplophora glabripennis*. The tree is constructed using the neighbor-joining method based on the full-length protein sequence alignments. Bootstrap analyses of 1000 replications are carried out, and bootstrap values > 60% are shown on the tree.

The sequences of vATPasea proteins in different insects were highly conserved. It contained V_ATPase_I structural domain (Figure 1A). The phylogenetic dendrogram of vATPasea proteins from 12 species was built to assess the evolutionary relationships (Figure 1B). These sequences were from four Lepidoptera, *Pieris rapae*, *H. armigera*, *Bombyx mori*, and *P. xylostella*; two Hymenoptera, *Apis mellifera* and *Solenopsis Invicta*; two Diptera, *Aedes aegypti* and *Drosophila willistoni*; two Hemiptera, *Nilaparvata lugens* and *Myzus persicae*; and two Coleoptera, *Leptinotarsa decemlineata* and *Anoplophora glabripennis*. The unrooted tree revealed that the vATPasea-like proteins of species from the same order were clustered together. Apparently, vATPasea from *P. xylostella* belonged to Lepidoptera subclade (Figure 1B).

### 3.2. The Expression Profiles of PxvATPasea

**Figure 2:**
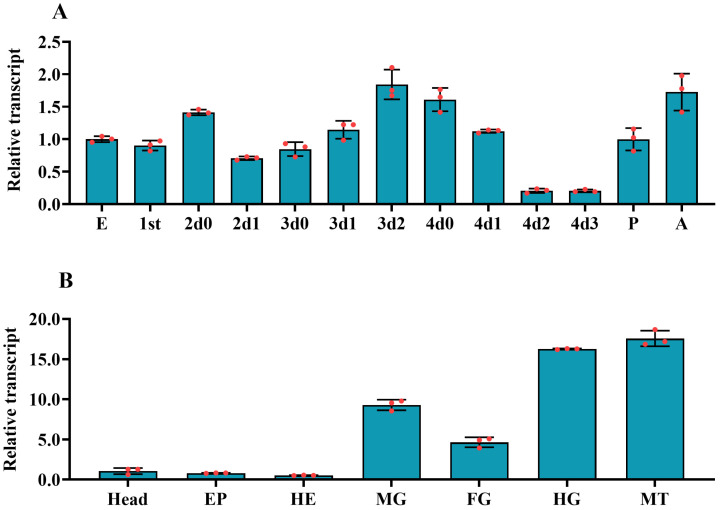
Temporal (**A**) and tissue (**B**) expression profiles of *PxvATPasea* in *Plutella xylostella.* For temporal transcription profile test, complementary DNA templates were derived from the eggs, first through fourth-larval instars at an interval of 1 day (1st indicated 0 and 1 days of first-instar larvae, D0 indicated newly ecdysed larvae or pupae, or D0 indicated newly ecdysed larvae), pupae (newly ecdysed pupae), and adults (newly emerged adults). For tissue expression pattern test, templates were from the head, foregut (FG), midgut (MG), hindgut (HG), hemolymph (HE), and epidermis (EP) of the day 4 fourth-instar larvae. For each sample, three independent pools of 5–10 individuals were measured in technical triplicate using qRT-PCR. The values were calculated using the 2^−ΔΔCT^ method. The lowest transcript levels at egg and head are set as 1. The columns represent averages with vertical lines indicating SE.

We used qRT-PCR to test the temporal expression pattern of *PxvATPasea*. The mRNA levels of *PxvATPasea* were widely transcribed during different developmental stages from embryo (egg) to adult. Its mRNA levels rose to a peak at day 2 of third-instar larval period; however, the lowest *PxvATPasea* level was detectable at the late fourth-instar larvae (day 2 and day 3). It was clearly observed that the mRNA levels of *PxvATPasea* regularly aggrandized during ecdysis period (Figure 2A).

The spatial expression profiles of *PxvATPasea* were evaluated in all tested tissues, including head, foregut, midgut, hindgut, hemolymph, Malpighian tubules, and epidermis. The mRNA levels of *PxvATPasea* were high in the hindgut and Malpighian tubules, intermediate in the foregut and midgut, and low in the hemolymph, epidermis, and head (Figure 2B).

### 3.3. Effects of RNAi for 800 ng of dsPxvATPasea at the Fourth-Instar Larvae

**Figure 3:**
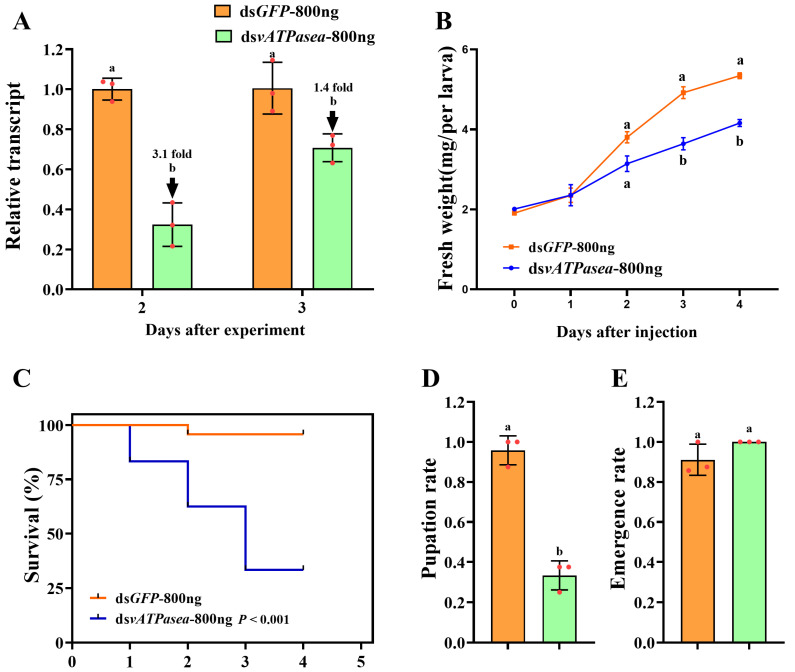
Effects of *PxvATPasea* silencing in fourth-instar larvae of *Plutella xylostella*. Newly molted fourth-instar larvae were injected with 0.2 µL of solution containing 800 ng of ds*PxvATPasea*, with an equivalent amount of ds*GFP* serving as the negative control. Injected larvae were subsequently transferred to fresh rape leaves for rearing. Expression levels of *PxvATPasea* were measured at 48 and 72 h post-injection (**A**). Relative transcript levels are presented as the ratio of relative copy numbers in treated larvae to those in the dsGFP control group (set as 1). Larval weight (**B**), survival rate (**C**), pupation rate (**D**), and emergence rate (**E**) were recorded over a 5-day trial period. Data are presented as mean ± SE. Different letters indicate statistically significant differences at *p* < 0.05.

To evaluate the effects of knockdown of *PxvATPasea* in *P. xylostella*, we silenced *PxvATPasea* by injection of dsRNA. Introduction of 800 ng ds*PxvATPasea* in the newly molted fourth-instar larvae significantly decreased the *PxvATPasea* mRNA level by 3.1 and 1.4 times at 2 d and 3 d, respectively (Figure 3A).

**Figure 4:**
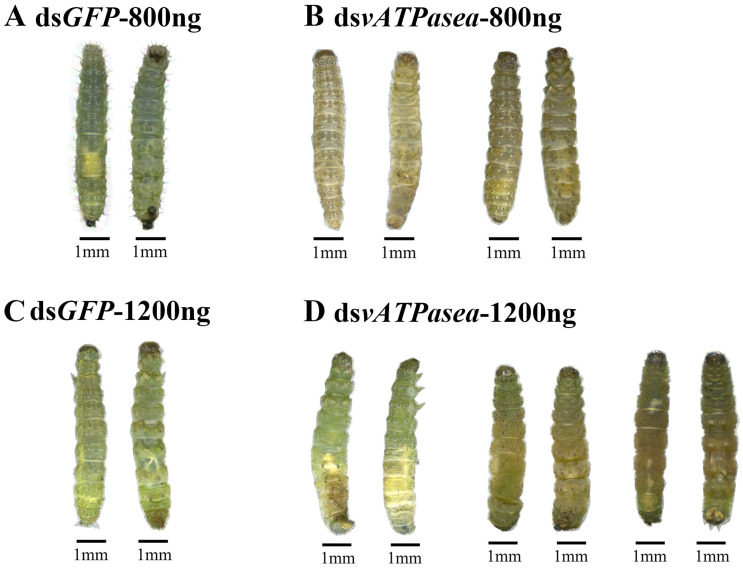
Impacts on knockdown of *PxvATPasea* in the fourth-instar larvae of *Plutella xylostella*. Phenotypic impacts of *vATPasea* silencing via dsRNA injection were assessed through developmental observation. Larvae were injected with solutions containing either 800 ng or 1200 ng of ds*PxvATPasea*, with an equivalent amount of ds*GFP* serving as the negative control. After injection, the larvae were transferred to rape leaves for rearing and monitored at 24 h intervals for developmental delays. Phenotypic documentation of both the ds*PxvATPasea*-treated and ds*GFP* control groups was performed on the third day after the start of the experiment.

Knockdown of *PxvATPasea* significantly restrained the development of the larvae, with fresh weights reduced by 26.0% and 22.1% at day 3 and 4, respectively, compared to the ds*GFP* control (Figure 3B). RNAi of *PxvATPasea* caused a significant mortality of the larvae. After the bioassay, mortality was 16.7%, 37.5%, and 66.6% at day 2, 3, and 4, respectively, in this treatment (Figure 3C). The remaining 33.4% larvae successfully pupated (Figure 3D). There were no significant differences in emergence rate among the two groups (Figure 3E). The ds*PxvATPasea*-treated larvae possessed small and misshapen body size. Subsequently, they slowly withered, blackened, and, lastly, died (Figure 4A vs. Figure 4B).

### 3.4. Impacts on Introduction of 1200 ng dsPxvATPasea at the Fourth-Instar Larvae

**Figure 5:**
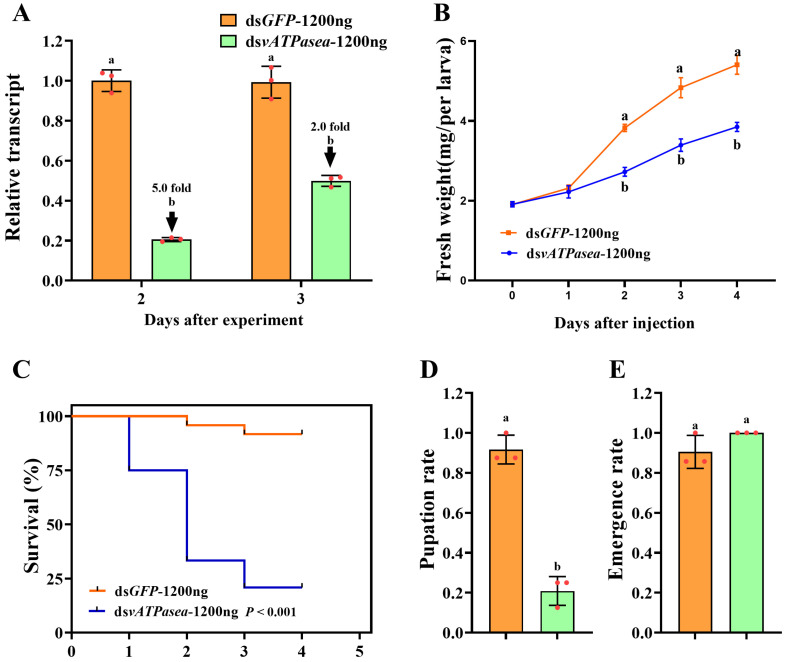
Effects of different doses of dsRNA on the suppression of PxvATPasea in Plutella xylostella. Newly molted fourth-instar larvae were injected with 0.2 µL of solution containing 1200 ng of dsPxvATPasea, with dsGFP injection used as the negative control. Under identical experimental conditions, PxvATPasea expression levels (**A**), weight (**B**), survival rate (**C**), pupation rate (**D**), and emergence rate (**E**) were analyzed. Data are shown as mean ± SE. Different letters denote significant differences at *p* < 0.05.

Introduction of 1200 ng ds*PxvATPasea* by microinjection at the newly molted fourth-instar larvae successfully reduced the mRNA level of *PxvATPasea* by 5.0 and 2.0 times at 2 d and 3 d, respectively, compared to the ds*GFP*-treated group (Figure 5A).

RNAi of *PxvATPasea* significantly inhibited the larval growth (Figure 5B), with fresh weights decreased by 28.0%, 30.0%, and 28.8% on day 2, 3, and 4, respectively, compared to the ds*GFP*-injected group (Figure 5B). In addition, silencing *PxvATPasea* seriously led to larval lethality, with mortality at 25.0%, 66.6%, and 79.2%, at day 2, 3, and 4, respectively (Figure 5C). The remaining 20.8% larvae successfully pupated (Figure 5D), and the emergence rate showed no significant differences (Figure 5E). The *PxvATPasea* RNAi larvae failed to molt their cuticle to become pupae. Their body size was small and abnormal, compared with the ds*GFP*-injected group. Subsequently, these stunting *PxvATPasea* hypomorphs became withered, dried, and blackened slowly and eventually died (Figure 4C vs. Figure 4D).

## 4. Discussion

Currently, relatively little attention has been given to genes encoding V0 subcomplex subunits. In the present study, we identified a vATPase subunit a gene in *P. xylostella*. We uncovered that silencing *PxvATPasea* affects the survival and growth of larvae.

### 4.1. RNAi Is a Powerful Tool to Manage P. xylostella

RNAi is one of the most important technologies in the last century [36], which is used as a powerful tool to study the functions of target genes or screen lethal genes for pest management in environmentally friendly and species-specific way [5,37,38,39,40]. Moreover, the development and application of biopesticides based on RNAi is a popular subject in pest management [41]. Up to date, great strides have been made in using genetically modified crops to control virous pests, including *A. lucorum* [10], *L. decemlineata* [42], *Adelphocoris suturalis* [43], *Sitobion avenae* [44], *M. persicae* [45], and so on. This evidence has strongly indicated that an RNAi-based strategy is viable to control pests.

The first step is to select suitable candidate genes for the development of RNAi-mediated control methods. Numerous studies have demonstrated that housekeeping genes such as *actins* [46]; cellular function genes like vacuolar ATPase [47]; energy metabolism genes, including *eukaryotic translation initiation factor 5A* [48]; and 20E signaling network genes like ecdysone receptors (*EcR*) [49,50,51] are known to be necessary for the growth of larvae. They can be developed as dsRNA insecticides or transgenic crops expressing dsRNA to control pests.

At present, RNAi of *PxPiwi* [14] and *PxTH* [15] in *P. xylostella* affected larval growth. Therefore, it seems very feasible to apply RNAi technology to control population of this pest.

### 4.2. PxvATPasea Is a Potential Candidate Gene to Control the Larvae of P. xylostella

In short, our results indicated that ds*PxvATPasea* is a potential candidate gene to control the larvae of *P. xylostella*. Firstly, *vATPasea* is a highly conserved evolutionarily ancient enzyme with the conserved domain of V_ATPase_I (Figure 1), which performs a wide range of functions in eukaryotic species [16,17,18,22,52].

Secondly, *PxvATPasea* was abundantly transcribed during different developmental stages, from embryo (egg) to adult. The expression of *PxvATPasea* was high at day 2 of the third-instar larval period and was low in the late fourth-instar larvae. Consistent with our results, the mRNA level of *HvvATPasea* was high in the third-instar larvae in *H. vigintioctopunctata*.

Thirdly, vATPases have an important role in nutrient absorption in the gut of various insects [19,53,54]. Our results showed that *PxvATPasea* was abundantly transcribed in the hindgut and Malpighian tubules in *P. xylostella*. Likewise, in *H. vigintioctopunctata*, the expression levels of *HvvATPasea* were highly expressed in hindgut [28].

Lastly, we uncovered that the introduction of ds*PxvATPasea* by injecting two different doses of dsRNA at the fourth-instar larvae significantly decreased the mRNA levels of *PxvATPasea* at different times, severely affected larval development, and killed 66.6% and 79.2% of larvae, respectively, under 800 ng and 1200 ng conditions (Figure 3 and Figure 4). In line with our result, in *D*. *melanogaster*, P-element insertions in *vha100-2* are lethal [27]; RNAi of *HvvATPasea* in the third-instar larvae of *H. vigintioctopunctata* led to 100% larval lethality [28].

Consequently, *PxvATPasea* is a potential candidate gene to manage by RNAi-based management strategies in *P. xylostella*.

Current functional studies on insect vATPases have primarily focused on subunits of the V1 complex, while research on V0 complex subunits remains relatively limited. Silencing of vATPase subunit a in the migratory locust (*Locusta migratoria*) specifically affects the midgut epithelium and causes high mortality, mainly by reducing the number of columnar epithelial cells during midgut development, which subsequently lowers nutrient transport efficiency and leads to reduced body weight [55]. Similarly, RNAi-mediated knockdown of the vATPase-a2 gene in the *Sogatella furcifera* impacts feeding behavior, resulting in significant changes in mortality, phenotypic defect incidence, and adult eclosion rate [56]. In Lepidoptera, research on the vATPase a subunit remains largely unexplored. Current evidence demonstrates that knockdown of vATPasea in the diamondback moth reduces body weight and causes high mortality, consistent with findings from RNAi targeting vATPasea subunits in other insects. However, the precise mechanisms through which it influences vital physiological processes require further investigation.

### 4.3. Enhancing RNAi Efficiency by Stabilizing dsRNA in Insects

Multiple factors influence RNAi efficiency, with dsRNA stability being a critical determinant. Naked dsRNA introduced into insects via injection or feeding is susceptible to degradation by nucleases present in saliva, hemolymph, and the gut, which partially accounts for the low RNAi efficiency observed in certain insect species. Particularly in Lepidoptera, nucleases identified as RNAi efficiency-related nucleases (REases) have been recognized [57]. Studies demonstrate that insect hemolymph and gut fluid contain double-stranded ribonucleases (dsRNases), which are considered a major limiting factor for RNAi efficiency due to their impact on dsRNA stability in bodily fluids. In insects, reducing dsRNase activity helps minimize intracellular dsRNA degradation and enhances RNAi efficacy [58,59].

Regarding dsRNA degradation prevention, encapsulating dsRNA with protective materials effectively preserves its integrity. The use of nanoparticle complexes to encapsulate dsRNA represents a novel delivery strategy for RNAi-based applications. These diverse complexes facilitate improved cellular uptake of dsRNA due to their high transduction efficiency and low cytotoxicity, while also reducing the risk of nuclease degradation under environmental conditions, such as temperature variations and medium changes [60]. The most extensively tested nanocarriers include chitosan, liposomes, star polycations (SPcs), layered double hydroxides (LDH), and guanylated polymers (GNP). The control efficacy of different vATPase subunits in *S*. *furcifera* was evaluated using star polymer (SPc) nanoparticles via spray-induced and nanoparticle-delivered gene silencing (SI-NDGS), which successfully reduced target mRNA levels and vATPase enzyme activity, while also assessing the environmental safety of nanoparticle-encapsulated dsRNA [56]. Three distinct nanoparticle-encapsulated dsRNA complexes significantly knocked down multiple genes, including VATPase, in *Earias vittella* [61]. Loading dsRNA of the VATPaseA gene onto layered double hydroxide (LDH) enhanced its environmental stability, leading to mortality in *Holotrichia parallela* larvae and disruption of their cuticle and midgut structures [62]. Therefore, further research on RNAi approaches targeting ds*PxvATPasea* will represent a promising strategy for managing diamondback moth populations.

## Data Availability

The original contributions presented in this study are included in the article/Supplementary Materials. Further inquiries can be directed to the corresponding authors.

## Supplementary Materials

The following supporting information can be downloaded at: https://www.mdpi.com/article/10.3390/insects16101054/s1, Table S1: A list of primers used for RT-PCR of the genes; Figure S1: A display of nucleic acid sequences of *PxvATPasea* from *Plutella xylostella*; Figure S2. On-target/off-target prediction and high-risk off-target regions of ds*vATPasea*.
