# Supplementary material for: RNAi of vATPasea Affects Survival and Larval-Pupal Development in Plutella xylostella
Source: Insects. 2025 Oct 16;16(10):1054. doi: 10.3390/insects16101054 (PMC12564257; doi:10.3390/insects16101054)
Supplement: Supplementary file 1 [file insects-16-01054-s001.zip › insects-3930197-supplementary.pdf]

Supplementary Data

Table S1. Primers used in RT-PCR, dsRNA synthesis and qPCR

Fragment name	Forward primer	Reverse primer
**RT-PCR**		
PxvATPasea	GGCCATGTTCAGGAGCGA	CGTCCTTGTCCTCCTGTTC
**dsRNA synthesis**		
ds*vATPasea*	TTCTCCTTATGGGTTGTTTCT	CAGCAGGCACAGGAACACC
ds*GFP*	TAATACGACTCACTATAGG	CCTATAGTGAGTCGTATTA
**qPCR**		
q*PxvATPasea*	ACTCGATCGTGCTGGAGTTC	TTCGTCAGTGGAGAAGGTGC
q*PxRPL32*	CCAATTTACCGCCCTACC	TACCCTGTTGTCAATACCTCT

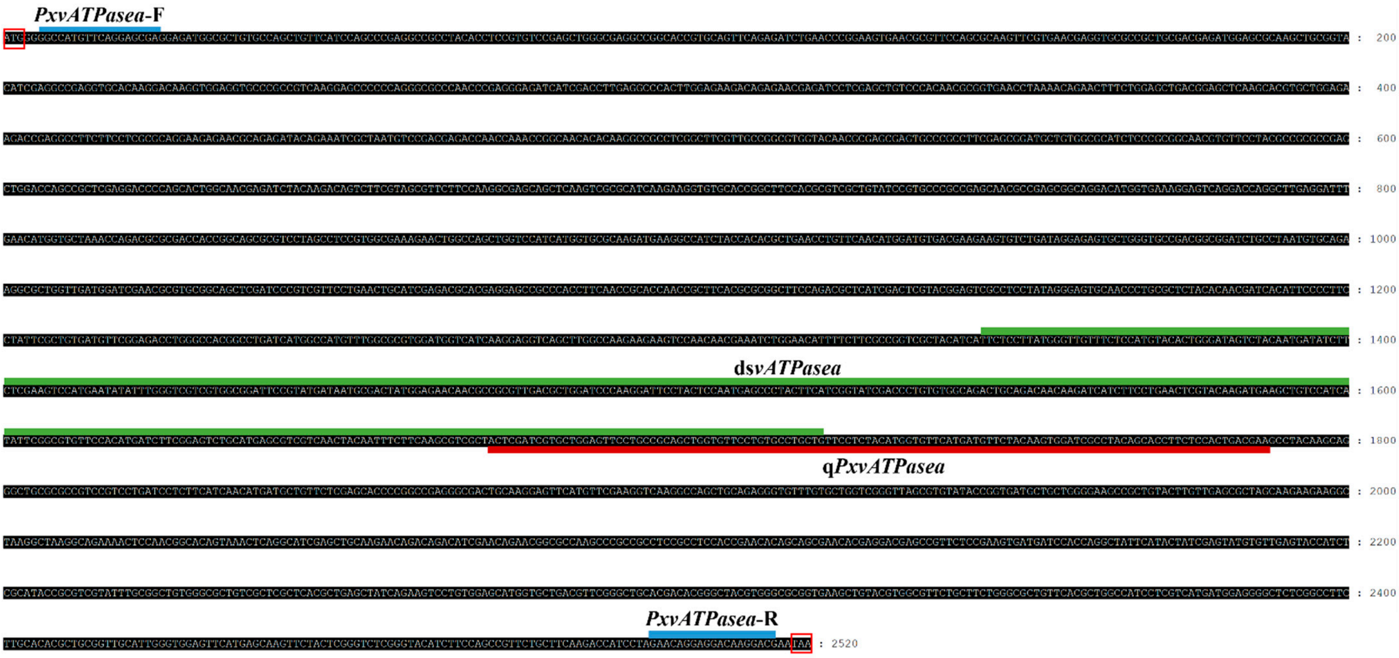

Figure S1. A display of nucleic acid sequences of *PxvATPasea* from *Plutella xylostella*. Start and stop codons are shown by red boxes. The primers for RT-PCR are indicated with green lines; the sequence for measuring the expression levels of *PovATPaseE* using qRT-PCR is marked with blue line; and the sequence for generation of ds*vATPaseE* is highlighted with red line.

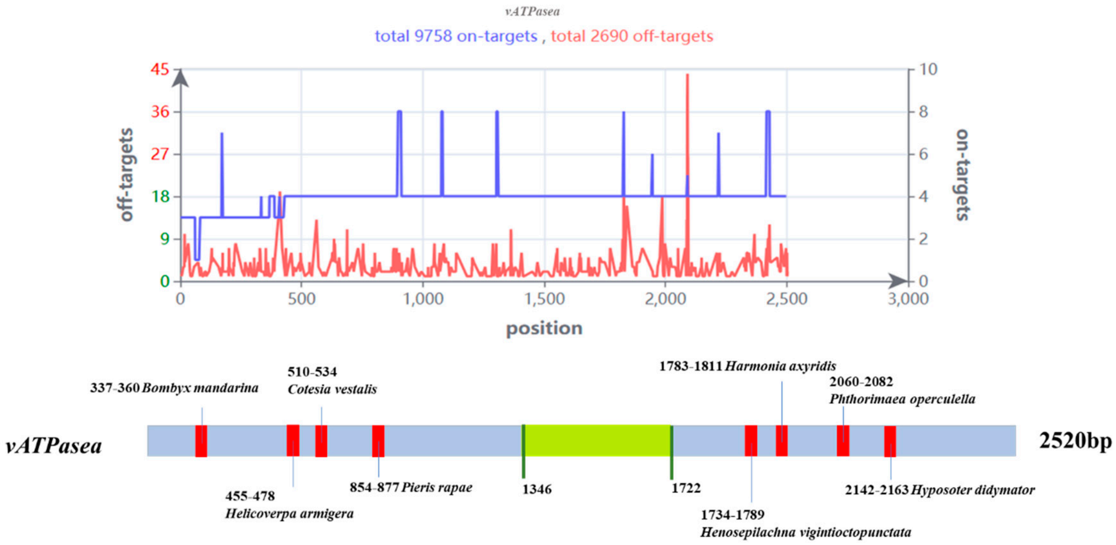

Figure S2. On-target/off-target prediction and high-risk off-target regions of *dsvATPasea*.An online tool(https://www.dsrna-engineer.cn) was employed to predict the quantity and positions of on-target and off-target sites for the *PxvATPasea* gene fragment during RNAi. The blue curve indicates on-target sites, while the red curve represents off-target sites. Predictions primarily considered eight arthropod species with ecological interactions (e.g., predation or phylogenetic similarity) to identify potential off-target regions in non-target organisms. The design of the dsRNA deliberately excluded these high-risk segments to minimize off-target effects.
